# Internet Gaming Disorders and Early Onset Psychosis in Young People: A Case Study and Clinical Observations

**DOI:** 10.3390/ijerph20053920

**Published:** 2023-02-22

**Authors:** Valerio Ricci, Domenico De Berardis, Giuseppe Maina, Giovanni Martinotti

**Affiliations:** 1 San Luigi Gonzaga Hospital, University of Turin, 10043 Orbassano, Italy; 2 NHS, Department of Mental Health, Psychiatric Service for Diagnosis and Treatment, Hospital “G. Mazzini”, ASL 4, 64100 Teramo, Italy; 3 Department of Neurosciences “Rita Levi Montalcini”, University of Turin, 10124 Torino, Italy; 4 Department of Neurosciences, Imaging and Clinical Sciences, Università degli Studi G. D’Annunzio Chieti-Pescara, 66100 Chieti, Italy

**Keywords:** internet gaming disorder, video games, psychotic disorder, early psychosis, first-episode psychosis, schizophrenia, psychosis

## Abstract

Background: Over the last ten years, the video game industry has grown exponentially, involving about 2.5 billion young adults in the world. The estimated global prevalence of gaming addiction has been reported to be 3.5% ranging from 0.21% to 57.5% in the general population. Moreover, during the recent COVID-19 pandemic period, school closures and stay-at-home measures have also further increased the opportunities for prolonged and intensified playing of video games. Little is known about the relationship between IGD and psychosis, and the literature is still scarce. Some characteristics of patients with psychosis, particularly those with a first-episode psychosis (FEP), may suggest that these individuals would be particularly liable to develop IGD. Case presentation: We report two cases of young patients with to Internet gaming disorder, experiencing early onset psychosis treated with antipsychotic therapy. Conclusion: Although it is difficult to show the specific mechanisms underlying the psychopathological alterations in IGD, it is clear that excessive exposure to video games could be a risk factor for precipitating psychosis especially in a vulnerable age group such as adolescence. Clinicians should be aware of the possibility of a higher risk of psychotic onset associated specifically with gaming disorders in very young people.

## 1. Introduction

In recent times, the Internet is a fundamental tool for communication, entertainment, study, and social relations [1]. This consistent evolution has also involved the world of video games, which have now become an important means of connecting with the rest of the world. Gamers can now be involved both socially and competitively with many other people, regardless of where they are. However, the loss of control of time spent on video games has revealed a number of negative consequences. Due to the strong impact it has on people’s mental health, this loss of control has been termed Internet gaming disorder (IGD) [2], and has been recently described as a problematic usage of the Internet with impulsive features [3].

Over the last ten years, the video game industry has grown exponentially, involving about 2.5 billion young adults in the world. The estimated global prevalence of gaming addiction has been reported to be 3.5% [4]. However, a recent literature review presented substantial variability ranging from 0.21% to 57.5% in the general population and from 3.2% to 91% in various clinical populations [5]. Moreover, during the recent COVID-19 pandemic period, school closures and stay-at-home measures have also further increased the opportunities for prolonged and intensified playing of video games, particularly among help-seeking individuals and others with gaming problems [6].

Moreover, the global pandemic has powered the growth of the influencer fanbase on social media and, with the increased time spent at home due to COVID-19 restrictions, has encouraged the growth of gaming influencers, known primarily for live streaming or posting videos of themselves playing video games and/or commenting on video games via social media. Influencers are people of high status on social media platforms who have a large follower base and are seen as dependable sources of information by their followers [7]. Influencers are used by marketers because they have demonstrated that they can positively impact their follower’s brand attitudes and product purchase [8]. Gaming influencers no longer attract a niche or fringe audience: some have loyal followers in the tens of millions. They are the most popular type of influencer followed by males aged 18–34, with almost a quarter (23%) of all adults in this demographic segment following gaming personalities.

The world around gaming influencers has increased the development of parasocial interaction, understood as symbolic, one-sided social connections that individuals imagine with media figures and celebrities [9]. This heterogeneous phenomenon not only has psychosocial but also psychopathological implications such as identity formation and autonomy from familiar members in adolescents, increasing the risk of developing psychosis.

Even before the official introduction of Internet gaming disorder in the fifth edition of the DSM and its recent revision [10], the scientific community debated whether to include gaming disorder within the broader issue of problematic Internet use or to focus on a more autonomous psychopathological condition. Historically, one starts talking about problematic gaming before the birth of the Internet. Some authors have described this phenomenon using a variety of terms such as “space invaders obsession” [11] and “computer catatonia” [12] or “video game addiction” [13], emphasizing the potential pathological use of videogaming from an early stage. Internet gaming disorder was introduced in 2013 in the the fifth revision of the Diagnostic and Statistical Manual of Mental Disorders as a condition requiring further studies. The term IGD unfortunately failed to fully clarify the ambiguities as this term would suggest that only Internet gaming should be taken into account, whereas excessive gaming may include offline gaming as well. Gaming addiction was finally recognized as an official diagnosis during the World Health Assembly in May 2019 and is now part of the 11th edition of the Classification of Diseases (ICD-10) [14] under the term gaming disorder. The disorder has been defined as a pattern of gaming behaviour characterised by: altered control over gaming, a priority given to gaming over other activities to the extent that gaming takes precedence over other interests and daily activities, and a continuation or escalation of gaming despite the occurrence of negative consequences. The behaviour pattern must be of sufficient severity to result in significant impairment of personal, family, social, educational, occupational, or other areas of functioning for at least 12 months. IGD has been associated with anxiety disorders, depression, suicidal ideation [15,16], behavioural disorders, social phobia, autism spectrum disorder, attention-deficit hyperactivity, obsessive-compulsive disorder, and personality disorders [17,18,19,20].

Little is known about the relationship between IGD and psychosis. The literature is still scarce and consists of case reports [21,22,23,24,25], two exhaustive reviews [18,26], and two cross-selectional studies. Some clinical features of patients with psychosis, especially those with a first-episode psychosis, make them particularly prone to the development of IGD. Hovewer, previous studies showed that over-gaming can induce neurobiological alterations commonly seen in other addiction diseases (activations of brain regions involved with reward; lower triggering in areas involved with impulse controls, motivation, cognitive support, and executive functions) [27,28] and enhanced dopamine levels similar to that elicited by psychostimulants [29,30]. These changes in brain function together with behavioural disorders associated with IGD, such as sleep deprivation [31], substance abuse [32], and other impairments, can be a key factor in the development of the onset psychotic episode in vulnerable individuals [33,34,35]. Indeed, all these conditions mainly present themselves in adolescence and young adulthood and are more frequent in the male population. Furthermore, both psychosis and Internet gaming disease have been associated with substance use, social isolation, and social anxiety disorders [36].

In order to demonstrate the role of gaming disorders in the development of psychotic symptoms, we describe two clinical cases of young people aged 15 and 16 who showed a clear picture of FEP, and then we provide a brief clinical insight on the co-occuring psychosis in IGD.

## 2. Case Report

### 2.1. Case One

She is a 15-year-old teenager. In the last two months, she has been reporting the emergence of paranoid symptoms such as the fear of being spied on in her room by cameras placed by her parents. She also reports the existence of secret devices that monitor her movements and act as a punishment if she makes a mistake. This symptomatology has worsened in the last few days to the point that the frightened parents have taken the girl to the emergency room. She started to game on the Internet around two years ago using the PC of her father who works as a computer scientist. Daily sessions usually lasted 6–8 h at a time, up to 12–14 h. She would mostly indulge in playing Massively Multiplayer Online Games with a strong emotional involvement and a tendency to be isolated. Although she continued to go to school, the patient led a withdrawn life, losing most of her friends; school performance had worsened considerably. Her mental status was characterised by a broad delusional atmosphere with persecutory thoughts towards her parents and medical staff. Her sleep decreased to only 4 h a day, along with decreased appetite and self-care. She also reported hearing a buzzing in her head. She was admitted to the hospital as she was unmanageable at home. She had no family history of psychiatric disorders. She also had no significant medical history. Blood tests and urine drug screening were in the normal range. The patient has never taken any kind of substance of abuse. She was treated with oral atypical antipsychotics (Aripiprazole 15 mg day) and lorazepam 2.5 mg before bedtime. The patient stabilized after 2 weeks and was discharged. She is currently undergoing follow-up in the outpatient department for 6 months, and is well maintained and functions scholastically. She continues taking aripiprazole 10 mg as the maintenance dosage.

### 2.2. Patient Two

He is a 16-year-old student. He came to our observation after the emergence of delusional persecutory ideation towards neighbours who are planning to kill him. The patient is convinced that they have implanted devices under his bed that film him during the night.

Psychotic onset occurred about three months ago with the presence of circumspect and suspicious behaviour, a tendency towards isolation, trouble going to school, and conflicts with teachers who were being accused of controlling him. He began playing video games on the mobile 3 years ago. Initially, he would play for 1–2 h a day. Gradually, the time spent gaming increased to 6–8 h a day extending up to 14 h on weekends. Gradually, there was an increased irritability when he was deprived of a PC by his parents. His scholastic performance also started declining. His sleep was reduced to 3 h; he often slept during the day and at night he engaged in video games. For two weeks, he started hearing a voice ordering him to stay safely at home because someone might damage him. He refused admission together with his parents, preferring to receive therapy at home, undergoing follow-up in the outpatient department for three times a week. He had no family history of psychiatric disorders. He also had no significant medical history. Blood tests and urine drug screening were in the normal range.

He was treated with oral atypical antipsychotics tablet Lurasidone 111 mg/die with improvement in psychotic symptoms. The patient is currently taking 74 mg of lurasidone as maintenance therapy undergoing follow-up in the Psychiatric Centre for 12 months.

The IGD Scale—Short-Form (IGDS9-SF) was used in both patients to assess the gaming behavior, while the positive and negative syndrome scale was used to assess the psychosis. The IGDSF-9-SF [37] consists of nine items, corresponding to the nine diagnostic criteria of the DSM-5 for Internet Gaming Disorder. Each item is rated from 1 (Never) to 5 (Very often). The scale addresses the degree of severity as well as the consequences of IGD by assessing the patient’s online and offline gaming activity over the past 12 months. The scale investigates the following domains: preoccupation, tolerance, withdrawal, reduction/interruption, loss of interest, continuous use, deception, escape, and conflict. A cut-off of ≥21 was established to distinguish IGD players (IGD+) from non-IGD players (IGD). In the validation study conducted by Monacis, the IGDS reports a Cronbach’s α index of 0.96, with an excellent internal consistency that makes it comparable to previously validated studies in England where the scale originates, Portugal, Spain, and Slovenia.

PANSS [38] is divided into the three subscales (i.e., positive, negative, and psychopathology) to evaluate psychotic symptoms. We evaluated positive symptoms and general psychopathology.

DES-II [39] was used to assess dissociative symptoms. This is the most common psychometric tool used for evaluating dissociative experiences. It is a self-administered questionnaire comprising 28 items based on the assumption of a ‘dissociative continuum’ ranging from a mild alteration to severe dissociation. Subjects are asked to select their choices for topics such as experiences of amnesia, absorption, depersonalisation, and derealisation.

The psychometric results are reported in Table 1.

All the patients and their parents gave informed written consent for the publication of this study.

## 3. Discussion

To date, this is the first clinical observation showing an early psychotic onset in young adolescents associated with gaming disorder.

In 1995, Ms Kimberly Young [40] had recognized that prolonged use of the Internet could have serious pathological implications and therefore coined the term “addictive use of Internet”. Over time, various terms have evolved: pathological Internet use, problematic Internet use, Internet use disorder, and pathological use of electronic media. Among Internet addiction online (online gambling, online chatting, online auction, and cybersex), gaming addiction is probably the most widespread, given the huge involvement of adolescents and children.

Given the scarce literature on the topic, it is difficult to show the specific mechanisms underlying the psychopathological alterations in IGD. Now, it is clear that gaming disorders are more frequent in the young male population, and strong correlations are shown with anxiety, depression, ADHD or hyperactivity symptoms, social phobia/anxiety, and obsessive-compulsive symptoms, while Internet addiction presents a strong correlation only with depression [41,42]. Furthermore, little is known about the prevalence of IGD and its consequences in patients suffering from a psychotic disorder. A previous study shows a positive relationship between psychotic-like experiences and problematic Internet use [43]. Neurobiological alterations typically observed in other addictions (reward deficiency, reduced impulse control mechanisms, impaired decision making, and impulsivity) [44,45] are the same as in IGD. Moreover, the involvement of the dopaminergic area (lower dopamine-transporter density and reduced occupancy of dopamine D2 receptors in the striatum) recalls certain neurobiological aspects peculiar to schizophrenia and continued use of the Internet can induce a decreased gray matter volume in specific areas of the brain [46].

As far as our clinical observations are concerned, we can deduce two mechanisms of psychotic development.

The first, more plausible for our young patients, is that the exposure to video games can trigger a psychotic process in previously vulnerable patients. This overexposure acts as a real psychostimulant, increasing the release of dopamine and activating the psychotic onset, with a mechanism that resembles what has been recently proposed for substance-induced psychosis [47]. On the other hand, we can speculate that prodromal symptoms of psychosis, such as social isolation, withdrawal from friends and family, tiredness, sleep disturbances, and feeling of disorientation, predict pathological gaming; in fact, these patients take refuge in a virtual reality where they could find a coping strategy to decrease their anxiety symptoms [48] and it helped them connect with others. This condition could contribute to the disintegration of subjectivity (often referred to as self-disorder), at a stage of life when the ‘self’ is built up. A core construct is that self-disorders are found in individuals at clinical risk of developing psychosis and that self-disorders predict the subsequent development of schizophrenic spectrum disorders [49].

A second theory is that video gaming worsens a pre-existing psychosis, sometimes masking psychotic symptoms. Indeed, some studies report that schizophrenic patients often find refuge in excessive video gaming as a way to escape reality and reduce stigma thus finding a secondary gain. This scenario paved the way for the possible use of video games for therapeutic applications, leading to positive outcomes such as affective involvement and renewed emotional activity [50,51]. However, it is difficult to determine the actual impact of gaming behaviour on patients’ health since gaming might enhance the social isolation observed in patients with FEP. Previous study proposes another pathogenetic mechanism: the development of psychotic manifestations induced by a sudden withdrawal of games [19].

The paucity of literature on this topic is striking and makes it difficult to draw clear conclusions. So far, we have only six case reports [21,22,23,24,25,52] and two cross-sectional studies [50,51,52,53]. In particularly, Chang comes to the conclusion that overexposure to Internet gaming is a coping strategy to mitigate self-stigma while Gauthier and colleagues try to determine the validity between two IGD scales in 235 patients aged from 12 and 17 years hospitalized in four psychiatry units, with the use of the IGDT-10, a self-reported scale, and the IDGT-P-10, an adapted scale developed to enable the patient’s parents to participate in GD screening by identifying the different symptoms presented by their child.

Some meta-analyses investigate the problem of Internet addiction both from an epidemiological point of view, where gaming addiction is most prevalent among adolescents in a population of 693,306 subjects including 113 epidemiological studies [54], and the worldwide prevalence of gaming disorders is 3.05% analysing 53 studies between 2009 and 2019 and 17 different countries [4]; and from a neurobiological point of view by selecting 40 studies utilizing a qualified whole-brain analysis, where the authors identified significant hyperactivation in the left striatum, right inferior frontal gyrus, and insula, and hypoactivation in the left superior frontal gyrus, left inferior frontal gyrus, and right precentral gyrus in IGS patients, confirming the critical role of the reward circuitry and executive control circuitry [55]. Other meta-analyses focused on the therapeutic perspective showing that pharmacotherapy combined with cognitive therapy and multi-level counseling might be an effective therapeutic strategy for youth with a gaming disorder [56], highlighting the sleep problem [57] and depressive symptoms [58]

Two important factors need to be considered. The first factor is the type of game: the *Massive Multiplayer Online Role Play Game* (MMORPG) is the most common type where the player creates a character (or ‘avatar’) in a fantasy world or other virtual setting alone or with other players who can be grouped in teams. All our patients played MMORPGs, which could be a risk factor for the development of GD and could even be associated with the onset of psychotic episodes: from a psychopathological point of view, the structure of these games is designed to be infinite, creating a strong sense of alienation leading to a psychotic onset [19]. Moreover, the structure of these games is designed to be infinite, so that even when players have completed the main objectives (growing in level, killing monsters, and fighting other users), there are additional objectives and alternative forms of “horizontal” progression (e.g., customization, and building and collecting virtual objects). For this reason, they have been defined as “never-ending games”, bringing about a temporal disconnect often found in psychotic pathology. The second factor is that partial dopamine agonist antipsychotics may increase the risk of developing a gambling addiction, thus confirming the hypothesis that dysregulation of dopamine levels may underlie both psychotic disease and gaming disorders [59,60].

Another interesting reflection focuses our attention on the relation between dissociation, increased in our patients, and IGD. Identification with an avatar establishes an awareness of surviving over time and space even in off-line mode, providing the preconditions for a disconnection between an ideal self and a real self. The principle of identity and continuity fails, leading to the development of psychotic manifestations as a possible compensation for this failure [61]. The full identification with the characteristics of the game also induces in some players a separation from their bodily self, thus reducing their ability to experience their bodily stimuli as well. From a phenomenological point of view, dissociative detachment (in terms of bodily disconnections) [62,63,64,65] has been regarded as a paradigmatic disturbance of embodiment and intersubjectivity perceived as lack of pre-reflexive self-awareness and disturbance in the process of intersubjectivity.

Environmental and personal factors in the genesis of the psychotic transition cannot be ignored as well: irregular sleep due to IGD was an environmental stressor that might have precipitated his becoming psychotic; moreover, we cannot exclude the possibility that the two adolescents may have had pre-existing ADHD that later evolved into a psychotic syndrome.

Finally, these case reports enhance the scarce literature on the issue of GD and psychosis in the initial stage.

Nonetheless, it is important to emphasise some limitations: The lack of longitudinal studies and the contradictory results obtained make it difficult to identify the relationship between IGD and psychopathology. The existing screening tools are not yet validated for psychotic disorders but for a general population and consist of self-reported scales based on the DSM-5 and ICD-11 criteria, which can be difficult to interpret even for patients without disabilities [66]. It is also important to take into account the type of video game and the personality profiles as a basis for future psychotic progression.

## 4. Conclusions

Clinicians should be aware of the possibility of a higher risk of psychotic onset associated specifically with gaming disorders in very young people. Today’s psychiatrists and psychologists should pay attention to the time spent by adolescents on the console, the knowledge of video games (multiplayer or solo), the quality of sleep, and the style of personality, and clearly pick up on elements of vulnerability triggering a psychosis. In the future, these elements would make it possible to establish an assessment scale not yet validated. A key issue to be supported is prevention. It might be useful to focus future attention on planning alternative activities to video games or on the development of small rules/advice where authority figures (parents or clinicians) can rely on upon noticing the first symptoms of the disorder. Therefore, parents should also be educated on the topic of gaming disorder so that they are ready to distinguish recreational use from pathological use and to recognise the first sign of it at an early stage of their children. At the same time, young people should also be instructed in the use of the console, so as to make them aware of the great advantages for a calibrated use and their great potential; on the other hand, it is crucial to remind them that they can also encounter negative effects in long-term use.

Finally, IGD is a complex clinical and social phenomenon that in recent years is becoming increasingly widespread, in view of the socio-technological changes affecting today’s society. Consequently, although, in recent years, several works of research have been conducted in this area, it is essential to direct empirical and clinical research in order to better know, evaluate, and treat this condition that not only involves younger people, but the entire society, given also the implications that this psychopathological condition has on the interaction of the individual with his or her social environment.

Prevention plays a key role in this regard in terms of primary (prevent the onset of a di ease), secondary (reduce the incidence of a disease), and tertiary (reduce the impact of a persistent health issue) prevention, emphasizing a public health perspective. Hence, there is a current scientific agreement on the need to develop well-controlled and methodologically solid evidence-based interventions. Evidence-based policies should be reliable and published in the media [67], concerning adequate school-based intervention programmes oriented primarily towards adolescents. These initiatives should ensure that specific online activities, such as problematic social media use [68] or gaming [69], should reflect the current state of knowledge, be theory-driven, and have the aim of enhancing skills and competencies associated with risk and protective factors. Yeun and Han [70], who performed a meta-analytic review on psychosocial treatment interventions that combined prevention initiatives, found large effects in reducing AI and the improvement of self-control and self-esteem, mainly in cases of parental-involvement counselling and self-control training programmes. Finally, the school system is increasingly used as a place to head prevention activities and to address health promotion and public health issues. This comes in the form of teacher and parent training, student education, and awareness-raising, which can increase protective factors and enhance positive behaviours or aspects of the environment that reduce the probability of negative events.

Future studies on a large clinical population are needed to provide further insight not only in understanding the neurobiological mechanisms of psychotic onset, but also in reaching a consensus about the definition, clinical status, and assessment of gaming addiction, in order to ensure appropriate and targeted prevention programmes involving researchers, teachers, and family members.

## Figures and Tables

**Table 1:** Scale scores.

	Patient 1	Patient 2
AGE	16	17
SEX	F	M
PANSS total score—initial visit	65	69
PANSS total score—final visit	45	36
PANSS Positive scale score—initial visit	25	22
PANSS Positive scale score—final visit	8	4
IGD-SF initial score	36	38
IGD-SF final score	15	19
DES II initial score	38	45
DES II final score	22	10
Self-reported/clinician-detected severe adverse events throughout the monitoring period	none	none

PANSS: Positive and negative syndrome scale, IGD-SF: Internet gaming disorder, SF: Short-form, DES II: Dissociative Experience Scale-II.

## Data Availability

The data presented in this study are available upon request from the corresponding author. The data are not publicly available due to privacy reason.
